# Perceptions and feelings of pregnant women undergoing outpatient follow-up for toxoplasmosis

**DOI:** 10.15649/cuidarte.3161

**Published:** 2024-05-22

**Authors:** Viviane Yumi Nakahara da Silva, Lucas Vinícius de Lima, Gabriel Pavinati, Gabriela Tavares Magnabosco, Nelly Lopes de Moraes Gil, Bianca Machado Cruz Shibukawa

**Affiliations:** 1 Universidade Estadual de Maringá, Maringá, Brasil. nakaharavivi@gmail.com Universidade Estadual de Maringá Universidade Estadual de Maringá Maringá Brazil nakaharavivi@gmail.com; 2 Universidade Estadual de Maringá, Maringá, Brasil. lvl.vinicius@gmail.com Universidade Estadual de Maringá Universidade Estadual de Maringá Maringá Brazil lvl.vinicius@gmail.com; 3 Universidade Estadual de Maringá, Maringá, Brasil. gabrielpavinati00@gmail.com Universidade Estadual de Maringá Universidade Estadual de Maringá Maringá Brazil gabrielpavinati00@gmail.com; 4 Universidade Estadual de Maringá, Maringá, Brasil. gtmagnabosco@uem.br Universidade Estadual de Maringá Universidade Estadual de Maringá Maringá Brazil gtmagnabosco@uem.br; 5 Universidade Estadual de Maringá, Maringá, Brasil. nlmgil@uem.br Universidade Estadual de Maringá Universidade Estadual de Maringá Maringá Brazil nlmgil@uem.br; 6 Universidade Federal do Mato Grosso do Sul, Três Lagoas, Brasil. bih.cruuz@gmail.com Universidade Federal do Mato Grosso do Sul Universidade Federal do Mato Grosso do Sul Três Lagoas Brazil bih.cruuz@gmail.com

**Keywords:** Toxoplasmosis, Pregnancy, Infectious Disease Transmission, Vertical, Toxoplasmosis, Congenital, Delivery of Health Care, Toxoplasmosis, Embarazo, Transmisión Vertical de Enfermedad Infecciosa, Toxoplasmosis Congénita, Atención a la Salud, Toxoplasmose, Gravidez, Transmissão Vertical de Doenças Infecciosas, Toxoplasmose Congênita, Atenção à Saúde

## Abstract

**Introduction:**

Toxoplasmosis persists as a neglected disease and poses a challenge to public health, especially due to the risk of vertical transmission, which can lead to countless biological complications for the newborn and to psychological and emotional repercussions for the mother.

**Objective:**

To understand the perceptions and feelings of pregnant women affected by toxoplasmosis undergoing outpatient follow-up.

**Materials and Methods:**

A qualitative and exploratory study developed with 12 women with gestational toxoplasmosis undergoing specialized outpatient follow-up in a municipality from the state of Paraná, Brazil. The data were collected through semi-structured individual interviews and subjected to content analysis, supported by descending hierarchical classification.

**Results:**

The pregnant women experienced situations ranging from diagnosis and treatment to preventing the disease in the child and family. These experiences generated fear, distress and uncertainty about the disease, which were not adequately addressed during prenatal assistance in primary care. However, the pregnant women emphasized the importance of the multiprofessional team at the secondary level in monitoring and health education.

**Discussion:**

Although the pregnant women felt confident about the treatment and its implications for the child's health, discovering the diagnosis impacted their everyday lives and those of their families, especially due to lack of reliable information about toxoplasmosis and to the absence of emotional support at the primary level.

**Conclusions:**

There was a temporary scenario of disinformation among these women, who were not properly guided and supported. However, the guidelines offered in secondary health care were essential for improving knowledge and practices in health.

## Resumen

**Introducción:**

La toxoplasmosis persiste como una enfermedad desatendida y representa un desafío para la salud pública, especialmente por el riesgo de transmisión vertical, que puede causar numerosas complicaciones biológicas para el bebé y repercusiones psicológicas y emocionales para la madre.

**Objetivo:**

comprender las percepciones y sentimientos de mujeres embarazadas afectadas por toxoplasmosis en atención ambulatoria.

**Materiales y Métodos:**

Estudio exploratorio cualitativo, desarrollado con 12 mujeres con toxoplasmosis gestacional en seguimiento en atención ambulatoria especializada en una ciudad del estado de Paraná, Brasil. Los datos fueron recolectados a través de entrevistas individuales semiestructuradas y sometidos a análisis de contenido, con apoyo de clasificación jerárquica descendente.

**Resultados:**

Las mujeres embarazadas vivieron situaciones que van desde el diagnóstico y tratamiento hasta la prevención de enfermedades en el niño y la familia. Estas experiencias provocaron miedo, angustia e incertidumbre sobre la enfermedad, que no fueron abordadas adecuadamente durante la atención prenatal en atención primaria. Sin embargo, las gestantes reiteraron la importancia del papel del equipo multidisciplinario secundario en el seguimiento y educación en salud.

**Discusión:**

Si bien las mujeres embarazadas se sintieron seguras sobre el tratamiento y las repercusiones en la salud del niño, el descubrimiento del diagnóstico impactó su vida diaria y la de sus familias, especialmente por la falta de información confiable sobre la toxoplasmosis y la falta de apoyo emocional en el momento de la gestación. nivel primario.

**Conclusiones:**

Hubo un escenario momentáneo de desinformación entre estas mujeres, quienes no fueron adecuadamente acogidas y orientadas. Sin embargo, la orientación en la atención secundaria es esencial para mejorar los conocimientos y las prácticas sanitarias.

## Resumo

**Introdução:**

A toxoplasmose persiste como uma doença negligenciada e representa um desafio à saúde pública, em especial pelo risco de transmissão vertical, podendo acarretar inúmeras complicações biológicas ao bebê e repercussões psicológicas e emocionais na mãe.

**Objetivo:**

Compreender as percepções e os sentimentos de gestantes acometidas pela toxoplasmose em acompanhamento ambulatorial.

**Materiais e Métodos:**

Estudo exploratório qualitativo, desenvolvido junto a 12 mulheres com toxoplasmose gestacional em seguimento na atenção ambulatorial especializada de um município do estado do Paraná, Brasil. Os dados foram coletados por entrevistas individuais semiestruturadas e submetidos à análise de conteúdo, com apoio da classificação hierárquica descendente.

**Resultados:**

As gestantes vivenciaram situações que perpassam desde o diagnóstico e o tratamento até a prevenção da doença na criança e na família. Essas vivências ocasionaram medo, aflição e incerteza a respeito da doença, os quais não foram devidamente acolhidos durante o pré-natal na atenção primária. No entanto, as gestantes reiteraram a importância da atuação da equipe multiprofissional do nível secundário no acompanhamento e na educação em saúde.

**Discussão:**

Embora as gestantes se sentissem seguras quanto ao tratamento e às repercussões na saúde da criança, a descoberta do diagnóstico impactou o cotidiano delas e de suas famílias, sobretudo pela falta de informações seguras acerca da toxoplasmose e pela falta de apoio emocional no nível primário.

**Conclusões:**

Houve um cenário momentâneo de desinformação entre essas mulheres, que não foram devidamente orientadas e acolhidas. Todavia, as orientações na atenção secundária foram essenciais para a melhoria dos saberes e das práticas de saúde.

## Introduction

Vertically transmitted (VT) diseases, which occur from mother to child during pregnancy, childbirth or breastfeeding, are still an obstacle^1^, including toxoplasmosis, which poses a threat to maternal and child health^2^. It is a zoonosis caused by a protozoan and transmitted horizontally, through contact with the agent, or vertically, which is one of the most serious and neglected routes of transmission^3^^,^^4^.

Toxoplasmosis causes significant morbidity and mortality, especially in developing countries with hot climates and/or weak infrastructure^2^. Worldwide, there are an estimated 190,000 cases of the congenital form every year^2^. In Brazil, the zoonosis has a high occurrence, which varies according to the region^5^; between 2015 and 2019, the country recorded 25 outbreaks of the disease, with five deaths^3^.

In the state of Paraná, toxoplasmosis has been compulsorily notifiable since 2003; in 2007 a field was added to identify pregnant women^6^. From that point until 2013, 1,147 cases of gestational toxoplasmosis and 79 cases of congenital toxoplasmosis were confirmed^6^. This context is highlighted by the fact that, in addition to the maternal repercussions, exposed children can suffer various complications, such as hydrocephalus, epilepsy, and loss of vision^2^.

Through the Paraná Mothers Network (*Rede Mãe Paranaense*, RMP), toxoplasmosis screening is recommended in all three trimesters of pregnancy, in order to achieve early detection of acute infection in pregnant women-with the aim of preventing transmission to the baby and, consequently, possible sequelae^7^. In addition, proper prenatal care can help ensure that, if affected, the child is asymptomatic or shows mild forms^2^.

It is recognized that the gestational process itself is permeated by feelings of fear and insecurity in the mother, including the possibility that the child will be born with some unexpected health condition^8^. Thus, the discovery of a possible illness in the child can have an emotional impact on the pregnant woman, conditioning negative perceptions and emotions towards the baby and herself, especially when there is a lack of information and professional support^9^.

A study carried out in a southern Brazilian municipality highlighted deficiencies in the quality of health care, both in primary and tertiary care, from the point of view of women with gestational toxoplasmosis^10^. In this sense, there is a need for research that considers other actors involved in prenatal care, such as secondary care, which is responsible for the shared follow-up of the mother child binomial in Paraná^7^.

Unveiling maternal experiences is necessary in order to understand how mothers deal with the discovery of the disease and the possibility of VT, with a view to contributing to increased care that takes into account not only biological demands, but also psychological and emotional ones. Therefore, this study sought to understand the perceptions and feelings of pregnant women affected by toxoplasmosis in outpatient care.

## Materials and Methods

This was an exploratory study with a qualitative approach. This methodological design aims to develop, clarify, and modify ideas and/or concepts in order to identify more precise problems and formulate hypotheses for further research^11^. The description of this research followed the recommendations set out in the Consolidated Criteria for Reporting Qualitative Research (COREQ).

The setting for this study was the medical specialties outpatient clinic at the University Hospital of Maringá (Hospital Universitário de Maringá, HUM), located in the northwestern region of Paraná. The HUM is a reference point for high-risk care in the state's 15th health region—with headquarters in the city of Maringá and covering 29 other municipalities in the surrounding area—in terms of maternal and child care, both on an outpatient and inpatient basis^7^.

Taking into account the severity of toxoplasmosis for the mother-child binomial, RPM stratifies pregnant women and children at high risk. During prenatal care at basic health units (BHUs), when a pregnant woman is diagnosed with toxoplasmosis, she is monitored by primary health care (PHC) and specialized outpatient care (SOC), with the aim of ensuring comprehensive and longitudinal care^7^.

Thus, the target population for this study was pregnant women with toxoplasmosis who were being monitored at the HUM outpatient clinic. The inclusion criteria were, as follows: having a confirmed diagnosis of toxoplasmosis recorded in medical records and being aged at least 18 years old. The pregnant women that were absent at the time of collection or who did not feel able to participate were excluded from the study.

The intentional sampling technique was used to select the participants, and the data was collected until theoretical saturation^11^^,^^12^. The main author of this study—a nursing student who had been trained to collect data—invited the pregnant women on site and introduced them to the research and its objectives. If accepted, the interview was carried out until the authors felt that unpublished data could not be added to the analysis.

The data were collected through individual interviews, in a private setting and at a single moment^11^, using a semi-structured script containing nine questions to answer: “which are the perceptions and feelings of pregnant women diagnosed with toxoplasmosis?”. The questions were developed by the researchers and adapted by three judges who are experts in the field, in order to avoid response bias^13^.

Data were also collected for characterization, namely: gestational age, age group, schooling, family income, occupational status, and marital status. The 12 interviews (nine main and three complementary to confirm saturation) took place between July and September 2022, and lasted a mean of 13 minutes each. It is noted that the researcher had no ties with the participants and there were no refusals to take part.

The interviews were audio-recorded and transcribed in full after collection using the double¬check technique, in order to guarantee the accuracy of the data^11^. Once this was done, the floating reading of the content analysis began, hypotheses were drawn up, and the corpus of the study was structured (pre-analysis)^14^. This material was then submitted to the Interface de R pour les Analyses Multidimensionnelles de Textes et de Questionnaires (IRaMuTeQ®) software program, version 0.7 alpha 2.

In IRaMuTeQ®, the material was explored using descending hierarchical classification (DHC), which categorized the text segments according to their vocabulary^14^^,^^15^. This set was presented in its lemmatized forms, already associated with each class (by means of the chi-square test with p<0.05), via a dendrogram. Each DHC class is made up of elementary context units (ECUs) which are similar within the class and different between them^15^.

From the colorful corpus of the DHC^15^, which allowed the extraction of fragments of the classes and their constituent elements, the data processing began, seeking to grasp the meanings of the categories and name them through thorough, critical and reflective reading^14^. Each class was illustrated by its ECUs—fragments from the testimonies. Absolute (n) and relative (%) frequencies were calculated for the variables characterizing the participants.

The dataset for this study, with the questions, the speeches and the colored corpus, was deposited in Mendeley Data^16^. In compliance with Resolution No. 466/2012 of the Brazilian National Health Council, approval was obtained from the Research Ethics Committee (opinion No. 5,453,990/2022). The participants agreed to the informed consent form. In addition, to ensure anonymity, they were identified as flowers.

## Results

When analyzing the characteristics of the 12 participants in this study, it was possible to see that the majority were women between the 14th and 27th gestational week, aged between 24 and 29, with (un)completed higher education, formally employed and married, whose family income was between two and three minimum wages (a reference to the value of one wage on the date of data collection: 1,212.00 Brazilian *reais*), as shown in Table 1.

**Table 1:** Descriptive measures corresponding to the sociodemographic and gestational characteristics of the study participants (n=12). Maringá, Paraná, Brazil, 2022

Characteristics of the pregnant woman	%(n)
Gestational age	
1-13 weeks	0.00(0)
14-27 weeks	66.67(8)
28-41 weeks	33.33(4)
Age group	
18-23 years old	16.67(2)
24-29 years old	58.33(7)
30-35 years old	16.67(2)
36-41 years old	8.33(1)
Schooling^a^	
(In)complete elementary school	25.00(3)
(In)complete high school	25.00(3)
(In)complete higher education	50.00(6)
Work situation	
Unemployed	33.33(4)
Employee	66.67(8)
Marital status	
Single	25.00(3)
Married	75.00(9)
Family income^b^	
Up to 1 minimum wage	33.33(4)
2-3 minimum wages	41.67(5)
4-5 minimum wages	25.00(3)

^*a*^ *sum of those who finished and did not finish the schooling level.* ^*b*^ *in Brazilian reais (reference of 1,212.00 per wage).*

**Figure 1:**
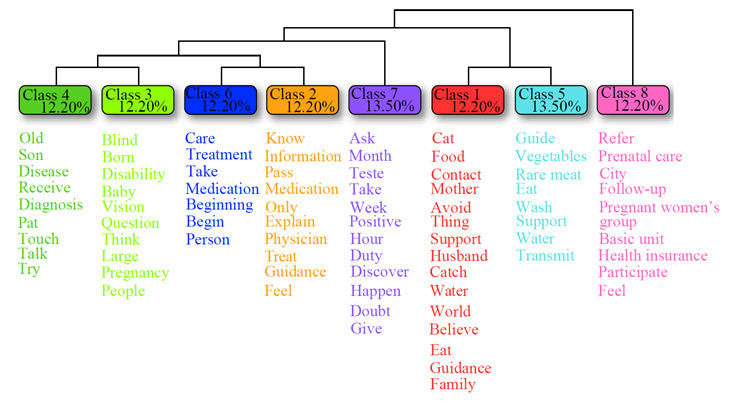
Dendrogram showing the descending hierarchical classification of the text corpus from the interviews (n=12). Maringá, Paraná, Brazil, 2022

*Note: The percentage represents the frequency of the class, composed of its elementary context units, in relation to the corpus.*

The DHC identified 2,904 occurrences of words, distributed in 340 active forms and divided into 84 ECUs, making use of 88% of the *corpus*. Eight classes were created, containing the most frequent words associated with each category (Figure 1). The interpretation of the classes follows the hierarchical order of the dendrogram (from right to left) and, in each class, the words closest to the top were the most recurrent within each category.

Class 8 (made up of the following main terms [p<0.05]: *refer, prenatal care, city, follow-up and pregnant women’s group,* and named **“Prenatal follow-up: weaknesses and strengths”**), referred to the importance of prenatal care from the participants' point of view, as well as to the absence of groups for pregnant women and the lack of guidance they received. This class can be represented by the following statements:

*I went to the BHU, but they didn't really give me much support, they referred me here [outpatient clinic]. I didn't join any support groups, nothing. (Clove)*

*I didn't have such good prenatal follow-up; I didn't join any pregnancy groups [...]. But I did receive guidelines on the tests and exams that are carried out during prenatal care, and I attend the appointments. (Hortence)*

Class 5 (made up of the following main terms [p<0.05]: *guide, vegetables, rare meat, eat and wash,* and named **“Preventive and protective care adopted by the family after the diagnosis”**), consisted of testimonies in which the pregnant women talked about the care they adopted, such as food hygiene, in order to prevent and protect their child and other family members. The class can be represented by the following statements:

*In fact, I've stopped eating rare meat, which I like a lot, and I've started taking more care when washing vegetables. (Clove)*

*[...] I've started taking more care, I dip [the food] in bleach and I'm avoiding eating rare meat. I don't eat out much; now I'm more attentive. (Daisy)*

Class 1 (made up of the following main terms [p<0.05]: *cat, food, contact, mother and avoid,* and named **“Maternal beliefs about the toxoplasmosis transmission means”**), revealed the pregnant women's knowledge about the transmission means of the disease and that this was acquired as a result of discovering the diagnosis. This category can be represented by the following statements:

*Well, I didn't know much before, you know. And then, after I saw that I was positive, I started researching and looking into things, and it's like a parasite, which is what you get through cat feces, eating poorly washed food, undercooked meat. (Daisy)*

*I couldn't explain it, I think it was some food, something I ate. Although I've had a cat all my life, my cats are house cats, [...] so I think it was something else, undercooked meat. (Tulip)*

In Class 7 (made up of the following main terms [p<0.05]: *ask, month, test, take and week,* and named **“Gestational exams: first contact with the diagnosis”**), the participants talked about the importance of undergoing gestational tests during prenatal care to screen for various infectious and contagious conditions, such as toxoplasmosis. This category can be represented by the following statements:

*I think we should [test] not only pregnant women. [...]. But the doctors could ask the adult to do it, because I didn't have any symptoms. If I hadn't done it, if I hadn't gotten pregnant and had prenatal care, I would never have known I had a disease. I could go blind without knowing it. (Tulip)*

*Now I have no doubts. In the beginning I had a lot, but I read many things. When I had my first trimester tests, they came back positive. I was scared at the time, because I did a lot of research and saw a lot of things that could happen. (Orchid)*

Class 2 (made up of the following main terms [p<0.05]: *know, information, pass, medication and only,* and named: **“First multiprofessional guidelines on toxoplasmosis”**) consisted of pregnant women's accounts of the guidelines given by health professionals during prenatal follow-up at the BHU. Below are some of the testimonies that represent this class:

*So, it's because I didn't feel anything, I don't know how to know what it is. The doctor even told me it was blood, but I didn't feel anything. Oh, I don't have any doubts, because people said that when the baby was born, you just had to take medication and it would go away. (Lavender)*

*It was the nurse who gave me this information, gave me some guidelines, and then I went to the doctor. It was quite shocking; I didn't know what to do at first. It can also affect vision and neurological development. (Hortence)*

Class 6 (made up of the following main terms [p<0.05]: *care, treatment, take, medication and beginning,* and named **“Medication and care: the start of an effective treatment”**) referred to the treatment adopted by the pregnant women for their new condition, given by the use of the medications prescribed by the physician and the adoption of the care practices they were instructed to follow. This class can be represented by the following statements:

*I started talking to various people and realized that it's quite common today and that there is a treatment. I started taking the medication. I was worried, desperate. When [my daughter] grows up, I don't have much information about what might affect her. (Orchid)*

*It's been a very difficult process, finding out and adapting to the care measures, but I don't think it's going to be that complicated, because I'm taking all the precautions and medications. (Hortence)*

Class 3 (made up of the following main terms [p<0.05]: *blind, born, disability, baby and vision*, and named: **“After all, how can toxoplasmosis affect my child's life?”**) portrayed the pregnant women's knowledge about the possible prognosis of their children. When analyzing the interviews, it was possible to notice that visual impairment was the most recurrent of the answers, as noted in the following statements:

*I have some doubts, especially if my baby's going to be born blind, something like that, because they had commented on it, but I don't know, I don't know if there's any way to find out. It creates certain insecurity in me, you know? (Hortence)*

*I've heard that the baby can be born without sight, right? That it can affect vision. The doctor explained more or less. I'm really worried. I'm still going to love my baby, but I'm worried about the care. (Jazmin)*

Finally, Class 4 (made up of the following main terms [p<0.05]: *old, child, disease, receive and diagnosis,* and named **“The disease of the past: receiving the diagnosis in the present”**) represented how toxoplasmosis, an old disease in the lives of these women, had repercussions in their present, with discovery of the diagnosis during pregnancy. This category can be represented by the following statements:

*She [the physician] said it was old, [from] when I first discovered toxoplasmosis; I have a 6-year-old son. Then she said that the disease was probably not active. (Daisy)*

*He [the physician] ordered an investigation and I took the IgG [immunoglobulin G] test. When I took it to the specialist, he said it was an old disease. I felt a bit bad because I thought it could be recent and, as I'm pregnant, it could affect the baby. But after the result came out, the doctor saw that everything was normal. (Lily)*

## Discussion

Understanding the feelings of pregnant women with toxoplasmosis revealed that these women have experienced countless situations, ranging from diagnosis and treatment to preventing the disease in the child and the family. These experiences caused fear, anxiety, and uncertainty about the repercussions of toxoplasmosis, especially in the children, but reiterated the importance of the multiprofessional team in health monitoring and education.

The **“Prenatal follow-up: weaknesses and strengths”** class was characterized by statements related to the prenatal care process and its importance for a safe pregnancy. It also addressed the difficulties faced by pregnant women when attending the BHU in terms of lack of support and advice on pregnancy, especially due to the absence of pregnancy groups.

Prenatal consultations are an essential stage during pregnancy and consist of welcoming and monitoring pregnant women and their partners. One of its purposes is to promote comprehensive care for the mother-child dyad^17^. It is important for the prevention and/or early detection of health conditions, both maternal and fetal, which favors the healthy development of the infants and aims at reducing the risks to the pregnant women^7^.

It is the health professionals' role to provide guidelines on the gestational process in line with their particularities and needs, with the aim of reducing the anxieties and insecurities that may arise during this period. However, certain lack of knowledge about toxoplasmosis on the part of pregnant women was identified. This gap was also noticed in a study which found that the professionals failed to provide guidelines on pregnancy^18^.

In this sense, it is worth pointing out that insufficiency of health professionals in the teams was evidenced. This situation can result in weak prenatal care, given the possible overload of the family health strategy (FHS) teams, which are responsible for proximal follow-up of the population they serve. Consequently, this can lead to difficulties promoting guidance actions during care^19^.

For the pregnant women, the importance of prenatal care lies not in the number of appointments, but in the quality of welcoming and follow-up. It is of utmost importance that the professionals recognize each woman's particularities and guides her in a didactic way, accompanying her to the tests, welcoming her complaints, and understanding her physical and psychological changes^20^. This requires training to understand weaknesses, fears and insecurities.

The pregnant women's group is considered an important tool for raising awareness and for monitoring prenatal follow-up. It represents a safe space for solving doubts and sharing experiences, feelings and scientific and popular knowledge^21^. However, when we analyzed the interviews with the participants in this study, we saw that the groups were absent from the BHUs, further undermining care quality.

This scenario may have been driven by the COVID-19 pandemic, as pregnant and puerperal women were considered a risk group until the 14th postpartum day. Due to the impact of the pandemic, care at the BHUs was interrupted; however, the Ministry of Health recommended that prenatal assistance be guaranteed, with spacing between appointments and a recommendation to use the hybrid modality^22^.

Since the pre-pandemic period, prenatal care had already been difficult for pregnant women to adhere to and, in the face of COVID-19, this scenario has worsened^23^. In view of this, it is of great importance to institute actions aimed at adherence to care, especially in the group of pregnant women, given the relevance of this strategy for the prevention, detection and treatment of health conditions that affect the dyad.

The **“Preventive and protective care adopted by the family after the diagnosis”** class was made up of statements referring to the main changes introduced after discovering the disease. Toxoplasmosis contamination occurs through the ingestion of cysts present in food and water, and it is a disease that is easily spread, especially among people with lower socioeconomic status or poor sanitation infrastructure^2^.

The consequences of congenital toxoplasmosis can be diverse, causing irreversible harms and/ or leading to fetal death^24^. This highlights the importance of primary prevention, which is carried out through guidelines on the need for prenatal care and serological screening, as well as on the transmission sources and care measures, such as washing hands, fruits and vegetables, cooking meat properly and adhering to the treatment^25^.

The **“Maternal beliefs about the toxoplasmosis transmission means”** class dealt with the pregnant women's perspectives on the ways in which the disease is transmitted. When asked about the definition, the predominant answer was that it was “the cat's disease”. It should be noted that there are other factors associated with infection: although cats are the definitive hosts, the external cycle contributes to spreading the disease^26^.

Cats have the oocyst in their feces, but they can only transmit toxoplasmosis for seven to ten days, during which time their body produces antibodies and eliminates the parasite, while the immune memory remains^26^. Thus, despite the fact that pregnant women associate cats as the main form of infection, they represent low epidemiological importance in transmission, and better health education for the population is essential^2^^,^^27^.

However, even health professionals and students have insufficient knowledge about toxoplasmosis, especially in relation to the various contamination modes, such as drinking contaminated water, for example^28^. This lack of knowledge can lead to poor quality information being provided to pregnant women during prenatal care, which undermines their ability to prevent and protect themselves.

The **“Gestational exams: first contact with the diagnosis”** class reiterated the importance of prenatal care, emphasizing the need to undergo diagnostic tests. Some pregnant women believed that if they had not become pregnant, they would never have discovered the disease. This category is directly linked to the **“First multiprofessional guidelines on toxoplasmosis”** class, which reiterated the role of the SOC team.

In this sense, it is extremely important to identify susceptible women, with a focus on prevention and diagnosis, as seen in **“Medication and care: the start of effective treatment”**. These strategies can reduce the toxoplasmosis VT rates, both in symptomatic and asymptomatic pregnant women^29^^,^^30^. In Brazil, the Unified Health System (Sistema Único de Saúde, SUS) plays a fundamental role in providing access to the treatment.

Finally, in the **“After all, how can toxoplasmosis affect my child's life?”** class, the pregnant women reflected about the repercussions on their children's lives. This class is linked to the **“The disease of the past: receiving the diagnosis in the present”** category, which dealt with the impact of late diagnosis on pregnant women, who expressed fear at discovering an “old disease”, that might have various implications for their lives and those of their children.

This scenario of anxieties can be due to the weakness of the care provided to these women in PHC^19^, given that they were not properly welcomed and guided in this study. In this way, the importance of popular and permanent health education strategies is reiterated, with a view to improving pregnant women's knowledge, based on the qualification and integration of professional practices in the care network and based on comprehensive and longitudinal care.

It is therefore essential that health professionals, especially those responsible for prenatal care, are better prepared in order to increase pregnant women's knowledge about transmission, serological tests, adherence to therapy and the possible prognoses of toxoplasmosis. There is also an urgent need to plan actions aimed at controlling this neglected disease and eliminating it as a public health problem.

It is believed that this study can contribute to disseminating information on the theme among health professionals and managers. The professionals can recognize toxoplasmosis as a serious infection and try to improve their knowledge about the disease and its management, in order to provide the appropriate guidelines to pregnant women during prenatal care, regardless of the complexity level of the service.

On the other hand, the managers can get closer to these women's perceptions and experiences and, based on this, consider the specificities required to propose more appropriate health actions and services, taking into account their emotional and psychological demands. In this way, it would be possible to seek to eliminate this condition as a public health problem and offer health services that are more empathetic and welcoming to women's emotional demands.

This study should be interpreted in the light of certain limitations, especially because it is restricted to a municipality with a health system that is possibly more favored in terms of supply, organization and access to services. Furthermore, the findings were limited to the perspective of women undergoing follow-up in the public sector; therefore, it was not possible to understand how private services deal with pregnant women's biological and psychological needs.

## Conclusion

This research managed to apprehend the perceptions and feelings of pregnant women with toxoplasmosis. It was revealed that a temporary scenario of disinformation persisted among these women, as they were not properly guided and emotionally welcomed during prenatal follow up in PHC. This context produced feelings of insecurity, fear and uncertainty about the possible complications and repercussions of the disease.

On the other hand, the guidelines received from the multiprofessional team at the SOC were essential for improving the pregnant women's knowledge and practices in health, enabling them to act in their homes through prevention and protection measures-with the aim of preventing spread of the disease and recognizing the need to adhere to the treatment as a strategy for controlling VT.
